# RUNX3 is down‐regulated in glioma by Myc‐regulated miR‐4295

**DOI:** 10.1111/jcmm.12736

**Published:** 2016-01-12

**Authors:** Xinxing Li, Jihui Zheng, Hongyu Diao, Yunhui Liu

**Affiliations:** ^1^ Department of NeurosurgeryShengjing Hospital of China Medical UniversityShenyangChina; ^2^ Department of RadiologyThe Fourth Affiliated Hospital of China Medical UniversityShenyangChina

**Keywords:** glioma, miR‐4295, RUNX3, N‐myc

## Abstract

MicroRNAs are increasingly reported as tumour suppressors that regulate gene expression after transcription. Our results demonstrated that miR‐4295 is overexpression in glioma tissues and its level is significantly correlated with clinical stage. We also found that miR‐4295 inhibited the cell G0/G1 arrest and apoptosis leading to promoted cell proliferation and activity. The murine modelling study revealed that female nude mice injected with U87/anti‐miR‐4295 exhibit subcutaneous tumours in the right groin. Compared with anti‐NC, the tumour volume was significantly decreased in anti‐miR‐4295 treatment group. Furthermore, we confirmed miR‐4295 mediates the expression of RUNX3 by targeting its 3′untranslation region. In addition, N‐myc protein also could bind to the promoter of pri‐miR‐4295 and inhibit the expression of RUNX3 in glioma cells. These results validate a pathogenetic role of a miR‐4295 in gliomas and establish a potentially regulatory and signalling pathway involving N‐myc/miR‐4295/RUNX3 in gliomas.

## Introduction

Gliomas are primary central nervous system (CNS) tumours accounting for almost 80% of all diagnosed tumours of the brain originating from brain parenchyma 1, 2. Glioma classifications made by World Health Organization grading scale (WHO, 2007) separates gliomas by cytologic features and degrees of malignancy after haematoxylin and eosin staining 3. They were further categorized into grades I, II, III and IV. Grade I are typically solid tumours such as pilocytic astrocytomas and subependymal giant cell astrocytomas. Astrocytoma, oligodendroglioma and oligoastrocytoma were divided into Grade II which tend to exhibit benign tendencies and portend a better prognosis for the patient 4. Anaplastic‐astrocytoma/oligodendroglioma (Grade III) always represents increased mitotic activity, while glioblastoma (Grade IV), accompanied by necrosis and micro‐vascular proliferation, exhibits increased the rate of mitosis 5. Currently, the WHO classification scheme is widely accepted and remains to be a practical and effective means to classify gliomas. However, this system is solely reflected by histologic visual criteria and prone to subjective inter‐observer variation 6, 7.

MicroRNAs (miRNAs), endogenous small non‐coding transcripts that regulate gene expression in post‐transcription, have been showed to play crucial roles in biological functions. The discovery of miRNAs has broadened our understanding of the mechanisms that modulate gene expression, adding entirely novel perspectives of regulatory control in the development of in tumour initiation, development and progression 8, 9, 10. Accumulating data showed that specific miRNAs were involved in human cancers such as lung cancer, breast cancer and astrocytic tumours 11. MicroR‐21 was the first miRNA investigated with glioma malignancy. Recent reports suggested that miR‐21 functions as an oncogenic miRNA by inhibiting the matrix metalloproteinase regulators RECK, TIMP3, heterogeneous nuclear ribonucleoprotein K, programmed cell death 4 and the tumour suppressor homologue of p53 (Tap63) 12, 13, 14, 15. Recent reports suggested that microR‐34a is down‐regulated in human gliomas and directly inhibits the expressions of c‐Met, Notch‐1 and Notch‐2 by binding to the 3′untranslation region (3′UTR) of target mRNA 16, 17, 18. In addition, miR‐326/Notch axis, shed light on the biology of Notch and can be used as a therapy approach in glioma in the future 19.

RUNX3 belongs to the Runt‐related gene family, whose members were all developmental regulators and had been shown to be involved in carcinogenesis 20. RUNX3 was shown to be down‐expressed in primary glioblastomas, and overexpression of RUNX3 in glioma cells resulted in significantly inhibited cell invasion and migration abilities 21. Accumulating evidence indicated that miR‐4295 may target USP28 in non‐small lung cancer and high expression of USP28 promoted tumour cells proliferation 22. However, there was no direct evidence indicated the potentially regulatory and signalling pathway involving miR‐4295/RUNX3 in gliomas.

Here, we focused the roles of N‐myc, miR‐4295 and RUNX3 and searched the potential signalling pathway involving miR‐4295/RUNX3 trying to elucidate the involved mechanism in gliomas. Our data showed that miR‐4295 targeted and subsequently repressed the expression of RUNX3, and overexpression of N‐myc promoted the level of miR‐4295 in glioma. Therefore, we proposed that miR‐4295, an N‐myc‐activated microRNA, could act as a unique oncogene by directly targeting RUNX3, which suggested that miR‐4295 may play a potential role in diagnostic and therapeutic.

## Materials and methods

### Clinical human glioma specimens and cell lines

Twenty paired human glioma and adjacent non‐tumourous glioma tissues were confirmed by pathological analysis and grouped into I to IV stage according to WHO standards. To detect the expression of miR‐4295, RUNX3 and N‐myc, RNA was isolated from the tissue samples according to the manufacturer's protocol.

The human glioma cell lines U87, U251, U373 and human brain glial cell lines (HBD) cells (control) were cultured in minimum essential medium‐a (MEM‐a) medium supplemented with 10% foetal bovine serum and 1% penicilin/streptomycin) in a humidified atmosphere at 37°C with 5% CO_2_. Transient transfection was performed with the Lipofectamine^™^ 2000 transfection reagent (Invitrogen, Carlsbad, CA, USA) according to the manufacturer's protocol. Each experiment in this study was performed at least three times.

### Reagents and vector construction

Anti‐miR‐4295 and miR‐4295‐mimics for detecting miR‐4295 were purchased from Applied Biosystems, Foster City, CA, USA. The siRNA‐N‐Myc, control siRNA were purchased from Ambion, Austin, TX, USA. Antibodies for RUNX3 (OMNI379) and GAPDH (07‐131) were from Enzo Life Science, Farmingdale, New York, USA and Millipore, Inc., Boston, MA, USA respectively. Two strands were annealed to clone a fragment of the RUNX3 3′UTR containing the target site of miR‐4295 with BamHI and XhoI sites. This construct was inserted into a BamHI‐XhoI digested Firefly luciferase reporter vector. To generate a miR‐4295 mutant, two strands were annealed and inserted into a BamHI‐XhoI digested RUNX3‐3′UTR‐mut reporter vector. The miR‐4295 promoter sequence was amplified by long‐PCR using long‐PCR with the PfuI polymerase and primers containing restriction sites (MluI or BglII) and then cloned into pGL3‐null reporter plasmid (Promega, Madison, WI, USA) to create pGL3‐pmiR‐4295.

### Dual‐luciferase reporter assay

The U87 cells were cotransfected with pcDNA3/miR‐4295 or anti‐miR‐4295 and the wild‐type or RUNX3‐3′UTR‐mut with the control in 48‐well plates. The luciferase fluorescent intensity was measured with an F‐4500 fluorescence spectrophotometer (Hitachi, Tokyo, Japan) 48 hrs after transfection, and firefly luciferase activity was normalized to that of Renilla luciferase. U87 and U251 cells were cotransfected with pGL3‐pmiR‐4295 and N‐myc inhibitors or control oligonucleotides. Firefly and Renilla luciferase activities were measured using the Dual‐Luciferase Reporter Assay System (Promega cat. no. E1910) 48 hrs after transfection, and Renilla luciferase activity was normalized to firefly luciferase activity.

### MTT assay

U87 and U251 cell lines, transfected with anti‐miR‐4295 or control, were plated in flat‐bottomed 96‐well microtitre plates at a density of 5 × 10^3^ cells/well respectively. 3‐4,5‐dimethyl‐2‐thiazolyl)‐2,5‐diphenyl‐2‐H‐tetrazolium bromide (MTT) assay was performed to evaluate cell viability. The culture was added with 10 μl of 5 mg/ml MTT agent and then incubated at 37°C for 4 hrs. After incubation, solubilization solution was added to stop the reaction followed 37°C overnight. The plates were read on a microplate reader (Sectramax 190; Molecular Devices Corp., Sunnyvale, CA, USA) at a wavelength of 590 nm.

### Colony formation assay

Tumour cells (5 × 10^3^) were plated in dishes and cultured at 37°C for a week and then stained with crystal violet for 20 min. and further photographed. Alpha Innotech (San Leandro, CA, USA) imaging software was used to quantify the number of cell colony.

### Real‐time PCR

Relative levels of mRNA were examined by qRT‐PCR using SYBR Premix Ex TaqTM (TaKaRa, Otsu, Shiga, Japan) according to the to the manual. Total RNA from tissues and cells was isolated using TRIzol reagent (Invitrogen) for mRNA analyses. RNA (10 ng) was first reversely transcribed into cDNA, which was used as template in the following experiment. After that, the PCRs were performed with iQ5 Real‐time PCR system (Bio‐Rad, Hercules, CA, USA). The PCR amplification protocol was as follows: 94°C for 4 min., followed by 40 cycles of 94°C for 30 sec., 58°C for 30 sec. and 72°C for 30 sec. in an. U6 small nuclear RNA (TaKaRa) was used as miR‐4295 internal control and GAPDH (Life Technologies, Carlsbad, CA, USA) was used as RUNX3 mRNA internal control.

### Western blotting

The cells (10^6^ cells) were lysed by Radio‐Immunoprecipitation Assay (RIPA) buffer (10 mM Tris‐HCl, pH 7.4, 1% Triton X‐100, 0.1% SDS, 1% NP‐40, 1 mM MgCl_2_) containing protease inhibitors after 48 hrs post‐transfection. The concentrations of protein were measured using a Micro BCA protein assay kit (Pierce Biotechnology, Waltham, MA, USA), and 50 g/lane of total protein was resolved on SDS‐PAGE gels and then transferred to a nitrocellulose membrane (Bio‐Rad). Blots were then blocked in PBS containing 5% non‐fat dry milk powder and incubated overnight at 4°C with the primary antibodies (1:3000). On the following day, the membranes were washed and incubated with HRP‐conguated goat antimouse secondary antibody (1:1000) for 2 hrs at room temperature followed by electrochemiluminescence (ECL) detection. After detection of protein bands, the blot was incubated with anti‐GAPDH antibody to confirm equal loading of the samples.

### Apoptosis and cell cycle assay

After transfection of 48 hrs, cells were harvested by trypsinization and washed with PBS. For apoptosis analysis, an Annexin V/7‐AAD Staining kit (Roche, Mannheim, Germany) was used according to the manufacturer's instructions. Apoptosis was analysed with fluorescence‐activated cell sorting (FACS) using the CellQuest software (Becton Dickinson, Bedford, MA, USA). Annexin V‐7‐AAD‐positive cells were regarded as apoptotic cells. For cell cycle analysis, the cells were firstly fixed with 70% ethanol at 4°C overnight. On the following day, fixed cells were washed with PBS, and then treated with RNase A (50 μg/ml) at 37°C for 20 min., further mixed with propidium iodide (50 μg/ml) for 30 min. in the dark. The stained cells were analysed with FACS by flow cytometry (FACSCalibur; Becton Dickinson). The phases of cells were quantified using the ModFit software (Becton Dickinson). A total of 10,000 events were counted for each sample.

### *In vivo* tumour growth

Female immune‐deficient nude mice (strain BALB/c nu/nu; 4–5 weeks old), were purchased from the animal centre of the Cancer Institute of Chinese Academy of Medical Science, and were bred at Department of Neurosurgery, Shengjing Hospital of China Medical University. Twenty‐eight female nude mice were divided into two groups, one was anti‐miR4295 and the other was anti‐NC (control). Suspensions of the stable anti‐miR‐4295 expressing cells or the control cells (1 × 10^7^ cells in 100 μl MEM‐α medium) were subcutaneously injected into female nude mice. Mice were monitored daily and two out of 28 mice died after injection of anti‐miR‐4295. Tumour volume was calculated on the basis of width (*x*) and length (*y*): *x*2*y*/2, where *x* < *y*.

### Statistical analysis

The data are presented as the mean ± S.D. The statistical analyses for the data comparisons were performed with a paired *t*‐test. *P* ≤ 0.05 was considered statistically significant (*P < 0.05).

## Results

### miR‐4295 is up‐regulated in glioma tissues and cells

**Figure 1:**
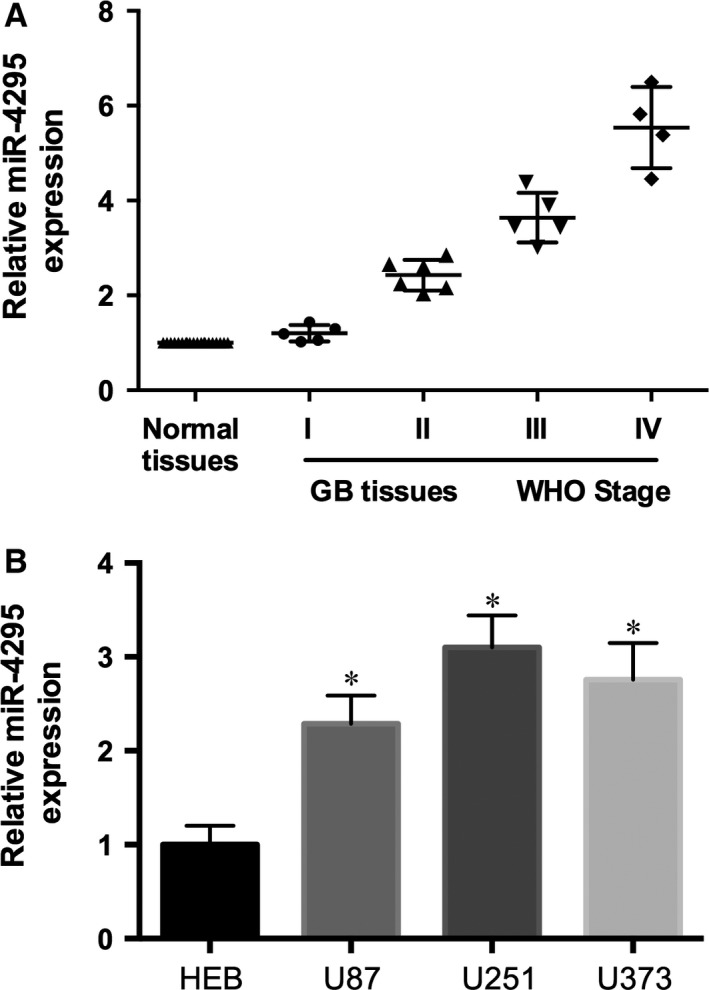
miR‐4295 is up‐regulated in glioma. (**A**) Expression of mature miR‐4295 in Normal tissues and GB tissues from stage I to IV (WHO standard). (**B**) Expression of mature miR‐4295 in U87, U251, U373 and HEB cells. *P < 0.05.

To determine whether miR‐4295 had an effect on the malignant phenotype of glioma, we performed real‐time PCR to detect miR‐4295 level of 20 paired clinical specimens. Compared with the adjacent non‐cancerous tissues, miR‐4295 was significantly up‐regulated in GB tissues (Fig. 1A). In addition, we found that the expression level of miR‐4295 was correlated with in tumour grade (according to WHO standards). Expression of miR‐4295 was also analysed in three human glioma cell lines U87, U251, U373 and HEB (Fig. 1B). Our analysis showed that miR‐4295 prominently overexpressed in high‐invasive glioma cell U373, whereas it was normal in HEB cells which was consistent with clinic. These data suggested that miR‐4295 may specifically correlate with the degree of tumour malignancy.

### miR‐4295 induces glioma tumourigenesis *in vitro* and *in vivo*

**Figure 2:**
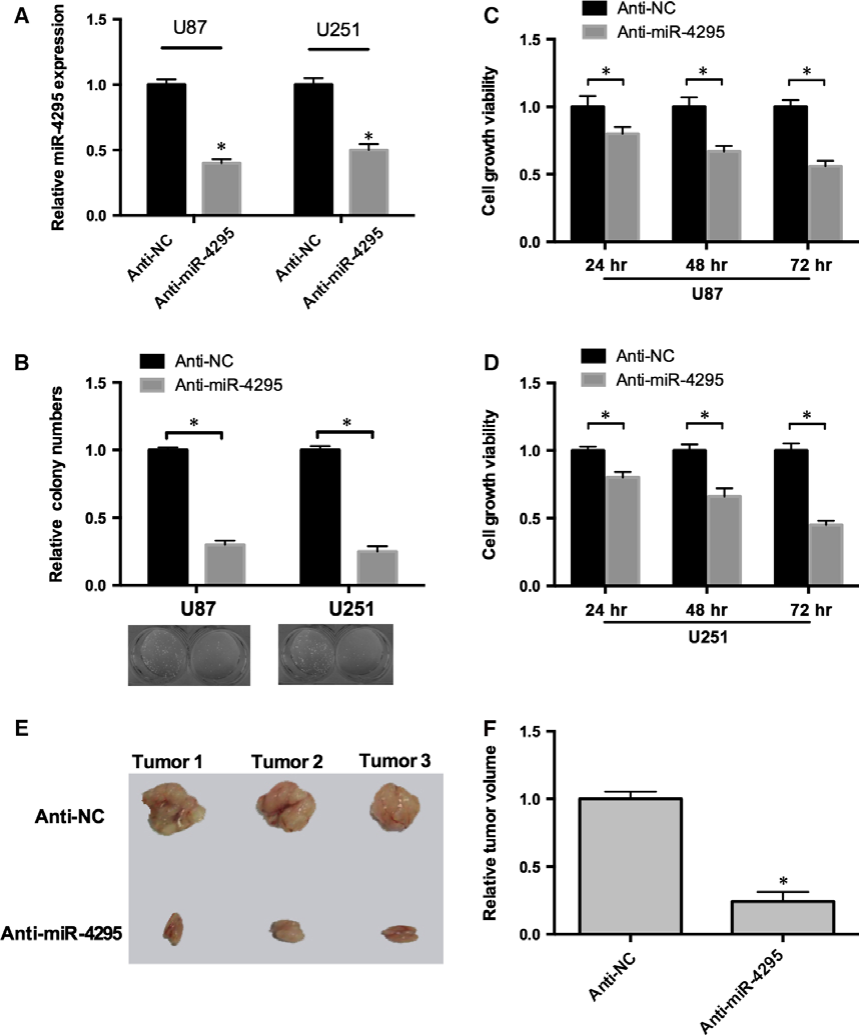
miR‐4295 induces glioma tumourigenesis. (**A**) Real‐time RT‐PCR of miR‐4295 in U87 and U251 cells infected with anti‐miR‐4295 or Anti‐NC. (**B**) The colonies formed in transfected U87 and U251 cells were stained with crystal violet at 8 days after transfection. (**C** and **D**) Cell viability was measured in both U87 (**C**) and U251 (**D**) cells 48, 72 and 96 hrs after transfection by the WST‐1 assay. (**E**) 3 typical tumour nodules in primary sites. (**F**) The tumour volume of 12 coupled mice were quantified. *P < 0.05.

To evaluate the significance of miR‐4295 overexpression in glioma cell, anti‐miR‐4295 and anti‐NC were transfected into human glioma cell lines U87 and U251. RT‐real‐time PCR results determined that the relative expression level of miR‐4295 expression in anti‐miR‐4295 was almost 60% (U87) or 50% (U251) lower compared with the anti‐NC respectively (*P* < 0.05; Fig. 2A). Cell colony formation results suggested that inhibition of miR‐4295 expression reduced the colony formation by 60% and 65% in U87 and U251 cell lines, respectively (Fig. 2B). Cell viability was measured in U87 and U251 cells up to 3 days after treatment. Cells infected anti‐miR‐4295 showed a significant decrease in viability compared with control. As shown in Figure 2C, cell viability decreased 17%, 20% and 50% at 24, 48 and 72 hrs after treatment in U87 cells. Consistent with the results in U87, miR‐4295 played a similar role in U251 cells (Fig. 2D) indicating that miR‐4295 may contribute to the growth of glioma.

To investigate the tumourigenesis of miR‐4295 in glioma *in vivo*, U87/anti‐miR‐4295 and control were transplanted into the nude mice. After 6 or 8 weeks, subcutaneous tumours were established in the right groin of 12 mice (two dead). Significant decrease in tumour volume was only observed in the anti‐miR‐4295 treatment group, and Figure 2E represented the typically tumours developed in mice. In all, these data indicated that miR‐4295 could promote tumourigenesis in glioma *in vitro* and *in vivo*.

### miR‐4295 inhibits cell apoptosis and regulates G0/G1 Transition

**Figure 3:**
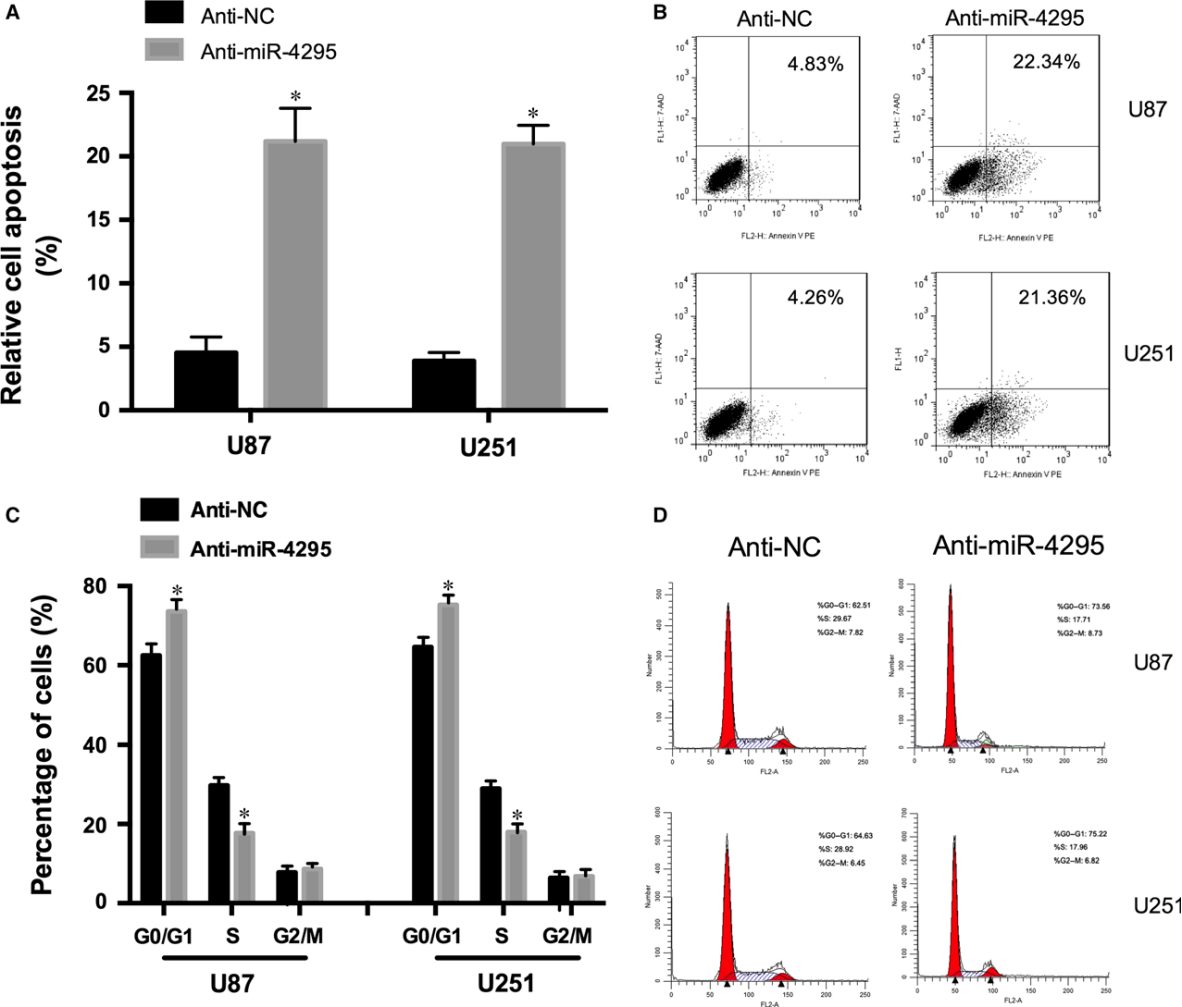
miR‐4295 regulates cell apoptosis and cell cycle. (**A**) Cell apoptosis of U87 and U251 cells infected with the anti‐miR‐4295 or anti‐NC. (**B** and **D**) FACS results of U87 and U251 cells infected with the anti‐miR‐4295 or anti‐NC. (**C**) Cell cycle analys of U87 and U251 cells infected with the anti‐miR‐4295 or anti‐NC. *P < 0.05.

To determine the functional contributions of miR‐4295 in the cell apoptosis, we knocked down miR‐4295 in U87 and U251. The cells infected miR‐4295 exhibited a significantly increased apoptosis (Fig. 3A and B) indicating that miR‐4295 may inhibit cell apoptosis in U87 and U251. To further investigate the effect of miR‐4295 on cell apoptosis, we transfected the anti‐miR‐4295 and anti‐NC into U87 and U251 cells respectively. The results informed knocking down miR‐4295 in both U87 and U251 cells significantly inhibited cell cycle progression.

### miR‐4295 directly targets RUNX3 and inhibits its expression

**Figure 4:**
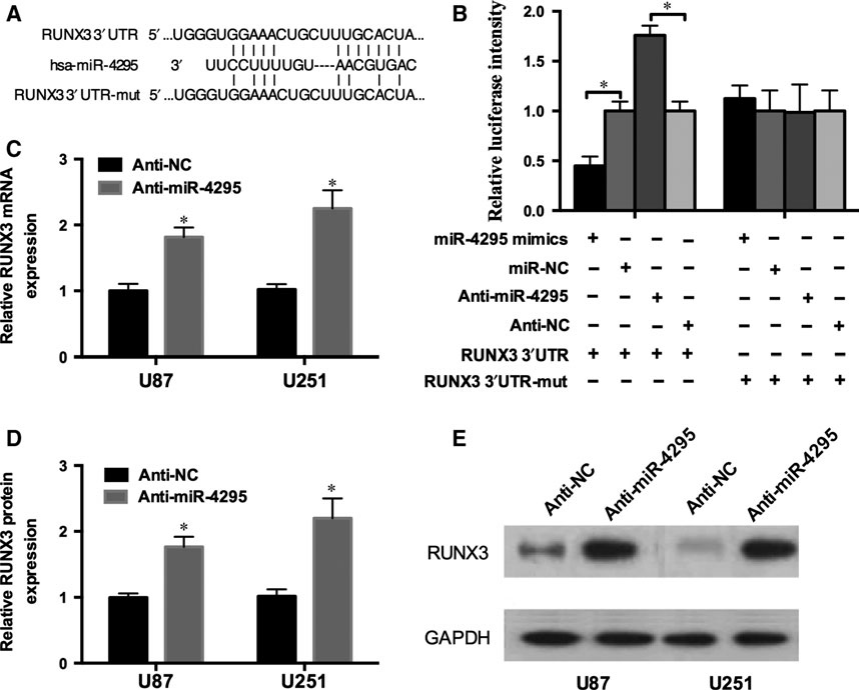
RUNX3 is the target of miR‐4295. (**A**) A schematic of the bioinformatics predicted seed region in the 3′UTR of RUNX3 as well as mutated 3′UTR. (**B**) Luciferase activity of the wild‐type or mutant RUNX3 3′UTR in U87 cells infected with the miR‐4295‐mimics/anti‐miR‐4295 and control. (**C**) Real‐time RT‐PCR of RUNX3 in U87 and U251 cells infected with the anti‐miR‐4295 or negative control. (**D** and **E**) RUNX3 protein expression analysed in U87 and U251 cells infected with anti‐miR‐4295 or control detected by qRT‐PCR (D) and Western blot (E). *P < 0.05.

To obtain direct evidence that RUNX3 is a direct target of miR‐4295, we generated a luciferase reporter construct (RUNX3‐3′UTR) and a mutant construct (RUNX3‐3′UTR‐mut) based on bioinformatics predictions (Fig. 4A). The luciferase fluorescence intensity of cells cotransfected with RUNX3‐3′UTR + miR‐4295 decreased 50%, however increased 1.7‐fold with RUNX3‐3′UTR + anti‐miR‐4295 (Fig. 4B). In addition, the luciferase fluorescence intensity was not significantly affected with RUNX3‐3′UTR‐mut (Fig. 4B) suggesting that the miR‐4295 directly targeted RUNX3‐3′UTR.

To further examine the role of miR‐4295 in the RUNX3 regulatory process, U87 cells were infected with anti‐miR‐4295 and anti‐NC. The expression of RUNX3 was measured by qRT‐PCR and Western blot. The transfection with anti‐miR‐4295 resulted in a significant increased expression of RUNX3 (84%) and RUNX3 protein by 2.15‐fold in Fig. 4D. In contrast, the anti‐miR‐592 induced a significant increase in LHCGR expression (Fig. 6A). Similar results were obtained in U251 cell lines. Thus, these results revealed that miR‐4295 recognizes and regulates RUNX mRNA through specific binding to its 3′UTR.

### RUNX3 was down‐regulated in glioma tissues

**Figure 5:**
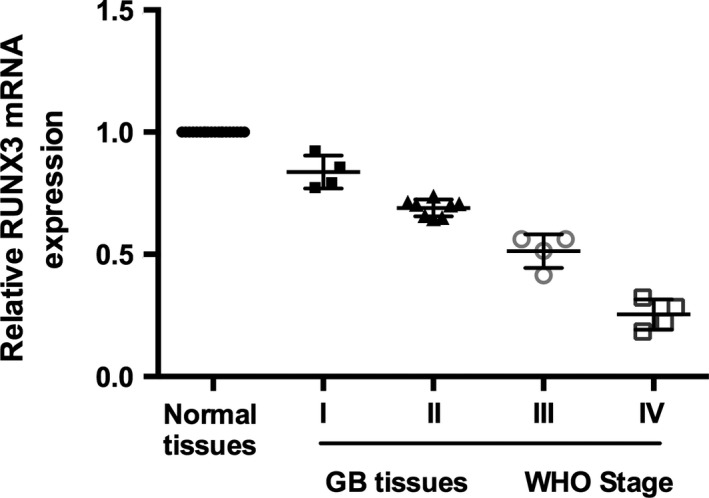
Inactivation of RUNX3 promotes glioma tumourigenic. Expression of RUNX3 in normal tissues and GB tissues from WHO stage I to IV (WHO standard).

Twenty pairs of human glioma tissues and their adjacent non‐cancerous tissues were analysed for RUNX3 expression by qRT‐PCR. The results showed that RUNX3 expression was generally inhibited in glioma tissues compared with the matched normal tissues (Fig. 5). Furthermore, we detected the expression level of RUNX3 from stage I to IV (WHO standard) and found that RUNX3 was most inhibited in IV stage tissues, whereas it was less in I stage tissues.

### N‐myc reduced RUNX3 expression in glioma cancer

**Figure 6:**
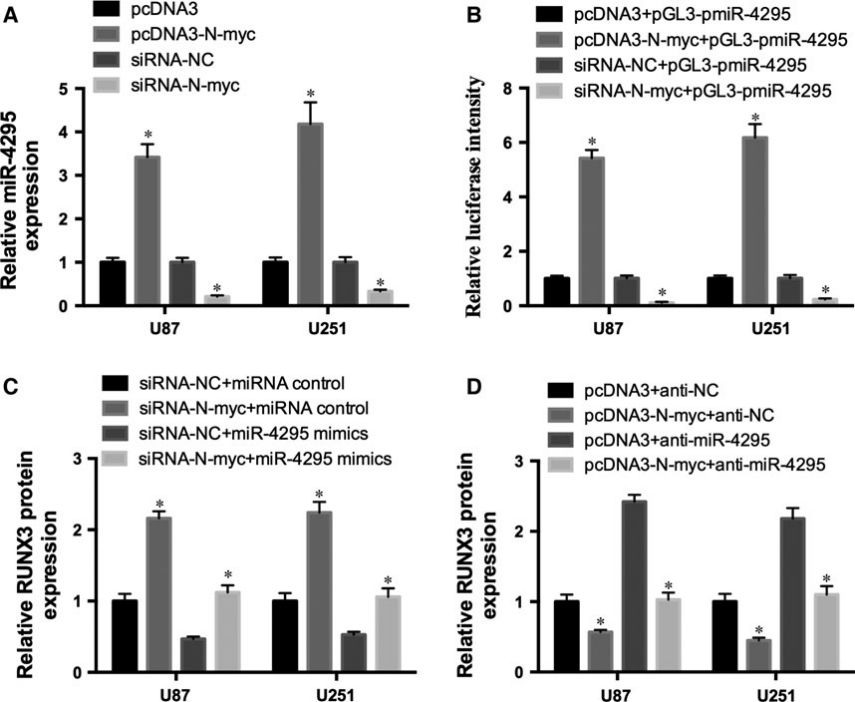
N‐myc inhibits RUNX3 expression. (**A**) Expression of miR‐4295 in U87 and U251cells transfected with pcDNA3‐N‐myc/pcDNA3, or siRNA‐N‐myc/siRNA‐NC. (**B**) Luciferase intensity of the pGL3‐pmiR‐4295 in U87 and U251 cells infected with the pcDNA3‐N‐myc/siRNA‐N‐myc and control. (**C** and **D**) RUNX3 protein expression of U87 and U251 cells cotransfected with miR‐4295 mimics/siRNA‐N‐myc (**C**) or pcDNA3‐N‐myc/anti‐miR‐4295 and controls (**D**). *P < 0.05.

To determine the exact function of miR‐4295 in glioma, UCSC genome browser was used. We found that N‐myc can potentially bind to the promoter of pri‐miR‐4295. Therefore, we transfected pcDNA3‐N‐myc or siRNA‐N‐myc into U87 and U251 cells and detected the expression of miR‐4295 in the cells. miR‐4295 expression was increased by overexpression of N‐myc and decreased by knocking down N‐myc (Fig. 6A). Moreover, pcDNA3‐N‐myc and siRNA‐N‐myc were co‐infected with pGL3‐pmiR‐4295 into U87 and U251 cells. The luciferase assay confirmed the results that pcDNA3‐N‐myc could promoted the luciferase activity, while siRNA‐N‐myc repressed the luciferase activity (Fig. 6B).

To further study the role of N‐myc in miR‐4295/RUNX3 axis, two loss‐of‐function rescue experiments were employed in U87 and U251 cells. Inhibition of N‐myc up‐regulated RUNX3 expression in glioma cells, however overexpression of miR‐4295 reversed the effect of N‐myc on RUNX3 expression (Fig. 6C). Similar results were obtained with transfecting pcDNA3‐N‐myc and anti‐miR‐4295(Fig. 6D). Moreover, inhibition of miR‐4295 was sufficient to restore RUNX3 expression repressed by N‐myc in glioma cells. These results indicated that N‐myc inhibited RUNX‐3 partly through binding to the promoter of pri‐miR‐4295.

## Discussion

miR‐4295 has been shown to directly target USP28, which may function as a tumour suppressor in non‐small cell lung cancer 22. Whether it affects the malignant behaviour of human glioma cells and is involved in the process of tumourigenesis remains unclear. To address these questions, qRT‐PCR assay was used to detect the miR‐4295 expression in human cancer tissues. The data showed that miR‐4295 overexpression is significantly correlated with clinical stage, and histological grading of the glioma cancer. The xenograft growth *in vivo* showed that miR‐4295 is involved in the process of tumourigenesis. We also found that miR‐4295 inhibited the cell G0/G1 arrest and apoptosis to induce glioma cell proliferation and activity.

In recent years, several molecular and epigenetic markers, such as isocitrate dehydrogenase 1 mutation, 1p19q co‐deletion, O^6^‐methylguanine DNA‐methyltransferase promoter methylation and epidermal growth factor receptor vlll (EGFRvlll) amplification, have been identified to predict tumour progression in glioma. Isocitrate dehydrogenase gene mutations, estimated to occur in 70–90% of diffuse lower grade gliomas, are strongly implicated in both tumourigenesis and prognosis 23, 24, 25. Loss of heterozygosity of 1p19q, was occurred in 60–80% of oligodendroglioma and up to 45% of oligoastrocytoma 26. The constitutively active mutant EGFRvIII, which is known as de2‐7 EGFR or DEGFR, is present in 25–30% of GBMs with concurrent EGFR amplification/overexpression 27, 28. O^6^‐methylguanine DNA‐methyltransferase promoter is frequently methylated in gliomas which represents as a positive prognostic marker that renders tumours more sensitive to radiation 29. Our aim was to investigate the genetic background of N‐myc/miR‐4295/RUNX3 and to identify a new marker that might play a role in gliomas behaviour.

RUNX3 has been identified as a tumour suppressor gene, which was widely distributed in human tumour, such as neuroblastoma, gastric carcinoma and small cell lung carcinoma, and breast carcinoma 30, 31, 32, 33. Prior reports have suggested that RUNX3 is frequently inactivated in human cancer cells and can be activated by hemizygous deletion of the RUNX3 gene, hypermethylation of the Runx3 promoter, or cytoplasmic sequestration of RUNX3 protein 32, 34. Furthermore, accumulating evidence demonstrated RUNX3 could inhibit the proliferation, tumourigenic and metastasis of glioma cells 21. Our results also showed that RUNX3 was repressed in glioma tissue, and was inversely correlation with the malignancy of glioma.

N‐myc proto‐oncogene occurs in approximately 20% of neuroblastomas and is associated with advanced stage disease, rapid tumour progression and poor prognosis 35. In addition, N‐myc induced miRNA signature in human cancer involving the activation and trans‐repression of several miRNA genes from paralogous clusters 35, 36. We suspected that miR‐4295 is regulated by N‐myc in glioma. To confirm this, we transfected N‐myc miR‐4295 into glioma cells (U87 and U251) and then examined the expression of miR‐4295. Our results suggested that N‐myc up‐regulated the expression of miR‐4295. Moreover, the luciferase assay results determined that N‐myc could bind to the promoter of the pri‐miR‐4295. Rescue experiment was carried out and these evidence conformed that overexpression of miR‐4295 rescued the effect of N‐myc on RUNX3 expression. Thus, we can get a conclusion that N‐myc mediated‐miR‐4295 inhibited RUNX3 expression in glioma.

In summary, our findings demonstrated that miR‐4295 was overexpression in glioma tissues and its level significantly correlated with clinical stage. Moreover, miR‐4295 mediates the expression of RUNX3 and effect proliferation, periodic blocking, and apoptosis of glioma cell. However, N‐myc protein also could bind to the promoter of pri‐miR‐4295, and inhibit the expression of RUNX3 in the glioma cells. In brief, these results may validate a pathogenetic role of a miR‐4295 in gliomas and establish a potentially regulatory and signalling pathway involving N‐myc/miR‐4295/RUNX3 in gliomas.

## Conflicts of interest

The authors declare no conflict of interest.
